# Prevalence of Respiratory Pathogens in Nasopharyngeal Swabs of Febrile Patients with or without Respiratory Symptoms in the Niakhar Area of Rural Senegal

**DOI:** 10.3390/pathogens13080655

**Published:** 2024-08-02

**Authors:** Dame Ndiaye, Georges Diatta, Hubert Bassene, Sébastien Cortaredona, Masse Sambou, Anna Julienne Selbe Ndiaye, Marielle Bedotto-Buffet, Sophie Edouard, Oleg Mediannikov, Cheikh Sokhna, Florence Fenollar

**Affiliations:** 1 Campus Santé Timone, Aix Marseille University, AP-HM, SSA, RITMES, 13005 Marseille, France; dame.ndiaye@ird.fr (D.N.); sebastien.cortaredona@ird.fr (S.C.); annajulienne.ndiaye@iressef.org (A.J.S.N.); sophie.edouard@univ-amu.fr (S.E.); cheikh.sokhna@ird.fr (C.S.); 2 IHU-Méditerranée Infection, 19-21 Boulevard Jean Moulin, 13005 Marseille, France; olegusss1@gmail.com; 3 EMR MINES, Campus Commun UCAD-IRD of Hann, IRD, Dakar 1386, Senegal; georges.diatta@ird.fr (G.D.); hubert.bassene@ird.fr (H.B.); massezorro1@gmail.com (M.S.); 4 Campus Santé Timone, Aix Marseille University, IRD, MINES, 13005 Marseille, France; 5 AP-HM, 13005 Marseille, France; marielle.bedotto@gmail.com; 6 Campus Santé Timone, Aix Marseille University, AP-HM, MEPHI, 13005 Marseille, France; 7 IRD, 13002 Marseille, France

**Keywords:** acute respiratory tract infections, SARS-CoV-2, adenovirus, human coronaviruses, influenza, RSV, *Streptococcus pneumoniae*, *Haemophilus influenzae*, Senegal, sub-Saharan Africa

## Abstract

Acute respiratory tract infections are one of the leading causes of morbidity and mortality worldwide. More data are needed on circulating respiratory microorganisms in different geographical areas and ecosystems. We analyzed nasopharyngeal swabs from 500 febrile patients living in the Niakhar area (Senegal), using FTD^TM^ multiplex qPCR and simplex qPCR to target a panel of 25 microorganisms. We detected at least one microorganism for 366/500 patients (73.2%), at least one virus for 193/500 (38.6%), and at least one bacterium for 324/500 (64.8%). The most frequently detected microorganisms were *Streptococcus pneumoniae* (36.8%), *Haemophilus influenzae* (35.8%), adenovirus (11.8%), influenza viruses (6.4%), rhinovirus (5.0%), SARS-CoV-2 (4.0%), and RSV (4.0%). The main microorganisms significantly associated with respiratory symptoms, with a *p*-value ≤ 0.05, were influenza virus (11.9% in patients with respiratory symptoms versus 2.9% in patients without), RSV (6.5% versus 2.6%), metapneumovirus (5.4% versus 1.3%), HPIVs (7.6% versus 1.0%), *S. pneumoniae* (51.9% versus 28.0%), and *H. influenzae* (54.6% versus 24.5%). Co-infections were significantly associated with respiratory symptoms (65.4% versus 32.9%). All the epidemiological data show a high level of circulation of respiratory pathogens among febrile patients, including those preventable by vaccination such as *S. pneumoniae*, raising the question of the serotypes currently circulating. Furthermore, the availability of affordable real-time etiological diagnostic tools would enable management to be adapted as effectively as possible.

## 1. Introduction

Acute respiratory tract infections are one of the leading causes of morbidity and mortality among children worldwide [1,2]. A variety of respiratory pathogens, including viruses and bacteria, can cause respiratory tract infections [3]. Common respiratory viruses causing infections are influenzas A and B, respiratory syncytial virus (RSV), human parainfluenza viruses (HPIVs), human coronaviruses (HCoVs), and adenoviruses (AdVs). Emerging novel respiratory viral microorganisms include swine influenza A H1N1, human metapneumovirus (hMPV), and human bocavirus (hBoV), particularly in pediatric patients, as well as SARS-CoV-2 [4,5,6,7]. The bacteria most frequently isolated from acute respiratory infections include *Streptococcus pneumoniae* and *Haemophilus influenzae* [8].

Childhood pneumonia is the single leading cause of mortality in children under the age of five. Most cases occur in India (43 million), China (21 million), and Pakistan (10 million), with additional high numbers in Bangladesh, Indonesia, and Nigeria (six million each) [9]. Each year, acute respiratory infections are responsible for 1.9 million deaths worldwide, 70% of which are in Africa [10,11].

In Senegal, acute respiratory infections remain the leading cause of infectious mortality [12]. In 1974, the country established a National Influenza Center [13], which has been included in the World Health Organization’s (WHO) global influenza surveillance network since 1996 [13]. The aim of influenza surveillance has traditionally been to detect influenza epidemics in the community at an early stage, to identify the predominant strains of influenza virus in circulation, and to issue public health recommendations. However, epidemiological data show that if only influenza viruses are monitored, a significant proportion of patients with influenza-like infections remained undiagnosed. For this reason, surveillance was extended to other respiratory viruses in 2012 [14]. Senegal’s syndromic sentinel surveillance network, known as the 4S network, is composed of primary health care centers (sentinel sites) located in various regions of the country (namely, Dakar, Dielmo/Ndiop, Fatick, Louga, Kaolack, Richard-Toll, Saint-Louis, Sokone, Tambacounda, Thies, and Ziguinchor) [14]. However, these sites do not cover the whole country, and studies focus exclusively on the detection of viruses—initially only influenza viruses, but increasingly other respiratory viruses too. Thus, recent data have shown a variety of viruses circulating within the community, although little information is available for bacteria [14]. However, respiratory tract infections are mainly treated empirically with antibiotics on the mere suspicion of bacterial infections, as they are considered one of the main causes of high morbidity and mortality rates [15]. Nevertheless, this strategy can potentially lead to the overconsumption of antibiotics and may have an impact on the susceptibility of bacteria to antibiotics in the future. In addition, the prevalence of different viruses according to the location of sentinel sites is generally not clearly identified. Senegal encompasses different ecosystems, and the circulation of microorganisms differs according to the location [14]. Even though it is not officially included in the 4S network, the city of Touba recently became another study area for respiratory infections during the Grand Magal, which is the largest Muslim religious gathering in West Africa, bringing together between four and five million Muslims each year [16]. In Senegal, there is a rainy season and a dry season, with a potential impact on the prevalence of microorganisms. During the rainy season, from June to October, the months are the hottest with temperatures ranging from 27 °C to 38 °C in coastal areas and greater humidity inland, while in the dry season, from November to May, temperatures oscillate between 22 °C and 30 °C during the day in coastal areas and are higher in inland.

The aim of this study, which began at the onset of the COVID-19 pandemic, was to characterize, for the first time in the Niakhar area in rural Senegal, the repertoire of respiratory pathogens, including bacteria and viruses, in febrile patients with and without respiratory symptoms.

## 2. Materials and Methods

### 2.1. Study Sites, Study Population, Sample Collection, and Ethics Statements

**Figure 1:**
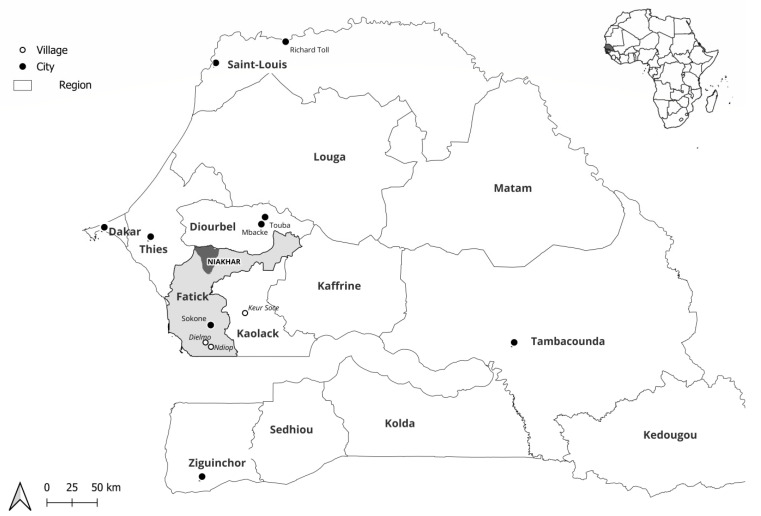
Location of the Niakhar area in rural Senegal (West Africa).

The study was carried out among the population of the Niakhar area, located in the Fatick region, 155 km southeast of Dakar, the capital of Senegal. We focused on this area because the circulation of respiratory pathogens has never been explored there before, although a population observatory has been in place for many years. The Niakhar area has approximately 48,000 inhabitants spread over 30 villages and covers an area of 230 km^2^. Febrile patients (defined by a corrected axillary temperature ≥ 38 °C) without or with respiratory symptoms (cough, sore throat, and/or rhinorrhea) consulting one of the four following health posts were included in the study: Diohine private, Diohine public, Toucar, and Ngayokheme (Figure 1). All were outpatients, and none were hospitalized.

The research protocol was approved by the Senegal National Health Research Ethics Committee (CNERS) as part of the long-term epidemiological surveillance of fever cases (declaration numbers #00087 and #00081). Signed informed consent was obtained from all febrile patients and/or their legal guardians in the case of minors. From the 2062 samples collected by nurses from January 2020 to December 2022, we empirically selected 500 samples (24.3%) to cover different age groups, different times of year, and different years. No formal sample size calculation or power analysis was performed. Instead, the sample size of 500 was mainly chosen based on cost considerations. The use of commercially available multiplex PCR kits targeting a wide range of respiratory microorganisms is expensive. As an indication, and knowing that kit prices vary from one country to another, we bought the FTD multiplex qPCR kit targeting 21 respiratory pathogens in Marseille, France, at EUR 1080 excluding tax (i.e., EUR 33.75 excluding tax per sample, for a kit allowing analysis of 32 samples). For this reason, we only analyzed a part of the collected samples. For a sample size of 500, the precision for an expected prevalence of 10% is ±2.63% and that for a prevalence of 5% is ±1.91%.

Of these 500 swabs, 270 (54.0%) were sampled from males and 230 (46.0%) were from females. In addition, 269 (53.8%) were from people aged between one month and five years, 141 (28.2%) were between the ages of 6 and 10, 50 (10.0%) were between the ages of 11 and 20, and 40 (8.0%) were aged 21 and over. All collected samples were placed in Σ-Virocult^®^ tubes (Medical Wire & Equipment, Corsham, UK) containing a preserving liquid medium. They were stored at −20 °C in the field and at −80 °C in the Dakar laboratory. They were then transported to Marseille (France) on dry ice and stored at −80 °C in the laboratory before processing. All 500 swabs were analyzed using multiplex and simplex real-time PCR tests.

### 2.2. Nucleic Acid Extraction

DNA and RNA were extracted from nasopharyngeal samples using a KingFisher instrument (Thermo Fisher Scientific, Illkirch-Graffenstaden, France) and a NucleoMag kit (Macherey-Nagel, Hoerdt, France) according to the manufacturer’s instructions. A total of 150 µL of each sample was extracted and eluted in 80 µL of elution buffer. An internal control was used to check the quality of the nucleic acid extraction.

### 2.3. Multiplex Real-Time PCR Assays

Multiplex real-time quantitative PCR (qPCR) assays were performed on a LightCycler^®^ 480 instrument (Roche Diagnostics, Meylan, France) according to the manufacturer’s instructions using the FTD^TM^ Respiratory Pathogens 21 kit (Fast Track Diagnostics, Esch-sur-Alzette, Luxembourg). Detection was carried using five tubes containing a mixture of primers and probes for different microorganisms, as well as an internal control. Tube 1 targeted influenza A (Flu A), influenza B (Flu B), influenza A subtype H1N1, and human rhinovirus. Tube 2 targeted human coronaviruses 229E (HCoV-229E), NL63 (HCoV-NL63), HKU1 (HCoV-HKU1), and OC43 (HCoV-OC43). Tube 3 targeted human parainfluenza viruses 2, 3, and 4 (HPIV-2, -3, and -4) and an internal control. Tube 4 targeted human parainfluenza virus 1 (HPIV-1), human bocavirus (hBoV), and human metapneumovirus (hMPV A/B). Tube 5 targeted respiratory syncytial virus (RSV A/B), human parechovirus (HPeV), enterovirus, and human adenovirus (HAdV). Around 5 μL of DNA/RNA extracts and 7.5 μL of mix were used for each of the five multiplex qPCR mixes. The program used for qPCR with the LightCycler^®^ 480 instrument was 50 °C for 15 min, 94 °C for one minute, 94 °C for eight seconds (40 cycles), and 60 °C for one minute. Samples were considered positive when the cycle threshold (Ct) value was <35.

### 2.4. Simplex Real-Time PCR Assays

Real-time simplex qPCR assays were performed on the CFX96 thermal cycler (Bio-Rad, Marnes-la-Coquette, France) according to the manufacturer’s instructions. This strategy was applied to identify bacteria such as *S. pneumoniae*, *H. influenzae*, *Staphylococcus aureus*, *Corynebacterium propinquum*, *Mycoplasma pneumoniae*, and *Streptococcus pyogenes* [17,18,19,20,21,22] (Supplementary Table S1) as well as the respiratory virus SARS-CoV-2, which was not included in the pools of microorganisms targeted by the FTD^TM^ kits.

Simplex qPCR amplifications of each sample were performed with a final reaction volume of 20 μL, including 5 µL of DNA extracts for bacteria or RNA extracts for SARS-CoV-2. More precisely, 3.5 μL of pure water, 0.5 μL of each forward/reverse primer, 0.5 μL of TaqMan probe, and 10 μL of Roche Mix (Taq Polymerase, dNTPs, and MgCl_2_) for bacteria and 8.5 μL of pure water, 0.5 μL of each forward/reverse primer, 0.5 μL of TaqMan probe, and 5 μL of Fast Virus for SARS-CoV-2 were used. Positive and negative controls were also added to each assay. The program used for qPCR with the CFX96 instrument for bacteria was 50 °C for two minutes, 95 °C for five minutes, followed by 40 cycles of denaturing, 95 °C for five seconds, 60 °C for 30 s, and finally, a cooling step to 40 °C for 40 s. The program for SARS-CoV-2 was 50 °C for five minutes, 95 °C for 20 s, 95 °C for 15 s (40 cycles), 60 °C for one minute, and 40 °C for 40 s. Samples were considered positive when the Ct value was ≤ 35.

### 2.5. Statistical Analysis

Categorical variables are presented as numbers and percentages. We used χ^2^ or Fisher’s exact test to estimate the association between categorical variables. Cramer’s V was also calculated to estimate the strength of association between the variables. Cramer’s V ranges from 0 to 1, where 0 indicates no association between the two variables and 1 indicates a perfect association between the two variables. Moreover, all possible combinations of microorganisms as co-infections were considered. All analyses involved two-sided *p*-values, with statistical significance defined by *p*  ≤  0.05. Statistical analyses were performed using SAS V.9.4 (SAS Institute, Cary, NC, USA) [23].

## 3. Results

### 3.1. Detection of Microorganisms

A microorganism was present in at least 366 of the 500 nasopharyngeal samples analyzed (73.2%) (Table 1). Viruses were detected in at least 193 of the 500 patients (38.6%), and bacteria in at least 324 of the 500 nasopharyngeal samples.

### 3.2. Detection of Viruses

Adenovirus, with a prevalence of 11.8% (59 patients), was the most frequently detected virus (Table 1 and Table 2). Following adenovirus, influenza A virus was the second most frequent with a prevalence of 6.2% (31 patients); twenty of these cases (64.5%) corresponded to influenza A H1N1 virus. The prevalence of rhinovirus was 5.0% (25 patients), followed by RSV and SARS-CoV-2, which had a prevalence of 4.0%, with 20 patients positive for each. The prevalence of metapneumovirus, HPIV-1, and bocavirus was 2.8%, 2.2%, and 2.0%, respectively. Common coronaviruses (HCoV-HKU1, HCoV-E229, HCoV-NL63, and HCoV-OC43), enteroviruses, parechoviruses, HPIV-2, HPIV-3, and influenza B virus were less frequently observed, with a low prevalence of less than 2.0%. Finally, HPIV-4 was not detected in any patient.

### 3.3. Detection of Bacteria

The two most frequent bacteria were *S. pneumoniae* and *H. influenzae*, with a prevalence of 36.8% (184 patients) and 35.8% (179 patients), respectively (Table 1 and Table 2). This was followed by *C. propinquum,* with a prevalence of 26.0% (130 patients). Two other bacteria, *S. pyogenes* and *S. aureus*, were also detected, but at a lower prevalence of 2.0% (eight patients) and 3.0% (14 patients), respectively. Finally, *M. pneumoniae* was not detected in any patient.

### 3.4. Co-Infection with Different Microorganisms

Co-infection was detected in 226 nasopharyngeal samples (45.2%, 226/500) (Supplementary Table S2). Overall, 113 of the 226 patients (50.0%) were co-infected with two pathogens. The combination of *S. pneumoniae* and *H. influenzae* was the most frequently detected (34.5%, 39/113). Among the double co-infections, two cases of SARS-CoV-2 associated with common coronaviruses, including HCoV-229E and HCoV-NL63, were also observed. Furthermore, 77 of the 226 patients (34.0%) were co-infected with three pathogens. The three most frequent triple co-infections were the following: (1) *S. pneumoniae*, *H. influenzae*, and adenovirus in 17 patients (22%, 17/77); (2) *S. pneumoniae*, *H. influenzae*, and *C. propinquum* in eight patients (8/77, 10.4%); and (3) *S. pneumoniae*, *H. influenzae*, and influenza A in six patients (6/77, 7.8%). In addition, 30 patients (13.3%, 30/226) were co-infected with four pathogens. The three most frequent quadruple co-infections were (1) *S. pneumoniae*, *H. influenzae*, *C. propinquum*, and metapneumovirus in three patients; (2) *S. pneumoniae*, *H. influenzae*, adenovirus, and bocavirus in two patients, and (3) *S. pneumoniae*, *H. influenzae*, adenovirus, and human rhinovirus in two patients. Five patients (2.2%, 5/226) were co-infected with five pathogens: (1) *S. pneumoniae*, *H. influenzae*, *C. propinquum*, SARS-CoV-2, and RSV; (2) *S. pneumoniae*, *H. influenzae*, *C. propinquum*, bocavirus, and HPIV-1; (3) *S. pneumoniae*, *H. influenzae*, *C. propinquum*, human rhinovirus, and enterovirus; (4) *S. pneumoniae*, *H. influenzae*, parechovirus, RSV, and HPIV-1; (5) *S. pneumoniae*, *H. influenzae*, *S. aureus*, *S. pyogenes*, and RSV. Finally, one patient was co-infected with six pathogens (*S. pneumoniae*, *H. influenzae*, parechovirus, RSV, HCoV-OC43, and human rhinovirus). Overall, in this study of the Niakhar population, there was no highly significant association between the coexistence of pathogens. While there is an association between RSV and parechovirus (Cramer’s V = 0.49) and between *H. influenzae* and *S. pneumoniae* (Cramer’s V = 0.47), all the remaining variables have very weak associations (Cramer’s V < 0.3). Moreover, as many associations have been evaluated, it is possible that these are random.

### 3.5. Association between Respiratory Symptoms and the Presence of Microorganisms

A total of 185 febrile patients consulting at the Niakhar health posts had respiratory symptoms, while 310 did not, and data were not available for five patients. Influenza A, including H1N1; RSV; metapneumovirus; HPIV-1; HPIV-3; bocavirus; *S. pneumoniae*; and *H. influenzae* were significantly (*p* < 0.05) associated with the presence of respiratory symptoms (Table 2). The presence of pathogens, including adenovirus, influenza B, rhinovirus, SARS-CoV-2, common coronaviruses (HCoV-229E, HCoV-NL63, HCoV-OC43, and HCoV-KU1), HPIV-2, enterovirus, bocavirus, parechovirus, *S. aureus*, *S. pyogenes*, and *C. propinquum*, was not significantly associated with the presence of respiratory symptoms. Finally, co-infections were significantly more frequently observed in febrile patients with respiratory symptoms than in those without (65.4% [121/185] versus 32.9% [102/310]; *p* < 0.001) (Table 3).

### 3.6. Seasonality

**Figure 2:**
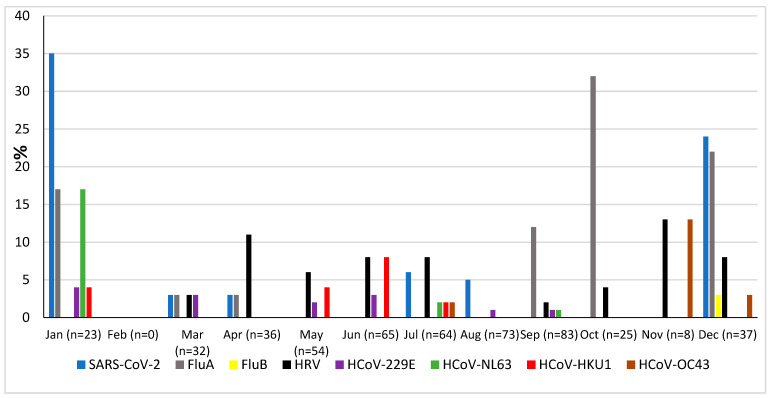
Seasonal prevalence of a first series of respiratory viruses (SARS-CoV-2, Flu A, Flu B, HRV, HCoV-229E, HCoV-NL63, HCoV-HKU1, and HCoV-OC43) from nasopharyngeal swabs in 500 febrile patients from the Niakhar area in rural Senegal (number of samples tested in brackets).

**Figure 3:**
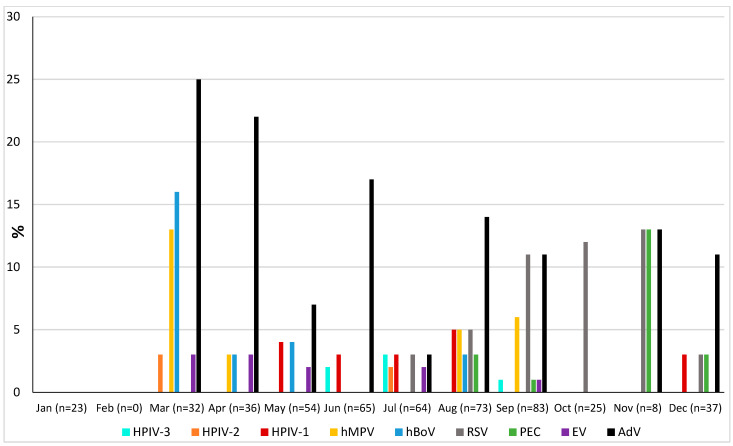
Seasonal prevalence of a second series of respiratory viruses (HPIV-3, HPIV-2, HPIV-1, hMPV, hBoV, RSV, PEC, EV, and AdV) from nasopharyngeal swabs in 500 febrile patients from the Niakhar area in rural Senegal (number of samples tested in brackets).

**Figure 4:**
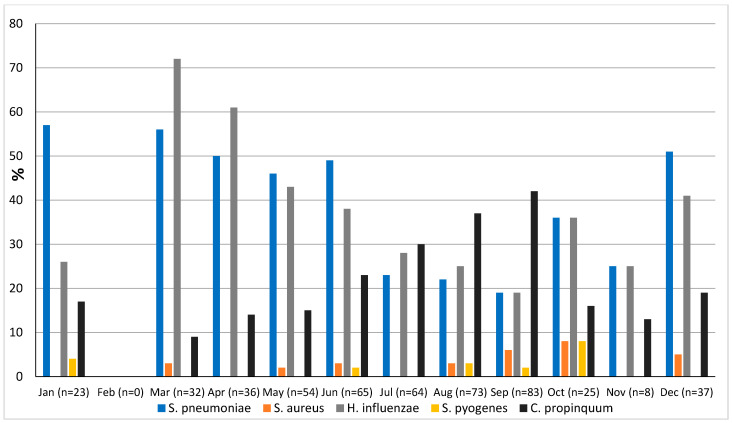
Seasonal prevalence of bacteria from nasopharyngeal swabs in 500 febrile patients from the Niakhar area in rural Senegal (number of samples tested in brackets).

The prevalence of microorganisms according to month and season is shown in Figure 2 and Figure 3. The highest peak of influenza A (including H1N1) was observed in October during the rainy season, and the other two highest peaks were observed in December and January during the dry season (Figure 2). The only case of influenza B was also observed in December. Except in the middle of the dry season (January and February), rhinovirus was generally observed throughout the year, with a higher peak at the end of the rainy season (November) (Figure 2). RSV was identified almost exclusively during the rainy season (Figure 3). Although SARS-CoV-2 seems to circulate during the different seasons, two peaks were observed in December and January (Figure 2). Among the common coronaviruses (Figure 2), HCoV-OC43 had the highest detection rate in November and December; HCoV-NL63 had the highest detection rate in January; HCoV-229E was identified sporadically throughout the year, with no distinct peaks; and HCoV-HKU1 was detected during both the rainy and dry seasons. Adenovirus was observed almost throughout the year (Figure 3). Bocavirus was mainly observed during the dry season, with a peak in March (Figure 3). Finally, *S. pneumoniae*, *H. influenzae*, and *C. propinquum* circulated all year round, though with peaks of varying amplitude depending on the season (Figure 4). *S. pneumoniae* and *H. influenzae* peaks were highest during the dry season. *C. propinquum* peaks were highest during the rainy season.

## 4. Discussion

Molecular analysis of 500 nasopharyngeal samples from 500 febrile patients from the Niakhar area revealed a positivity rate of 73.2% for at least one microorganism out of the tested viral and bacterial pathogens responsible for respiratory infections. This positivity rate was significantly higher in patients with respiratory symptoms (89%) than in those without (63.5%). This positivity rate is slightly lower than that reported in Keur Soce (95.2%) in central Senegal between August and December 2015 in patients with acute respiratory infection, for whom the detection of both bacteria and viruses was performed [24].

Adenovirus was the most frequently detected virus, with a prevalence of 15.3% in patients with respiratory symptoms and 10% in those without. It was also more frequently detected in younger people. While this prevalence is slightly higher than that observed in the villages of Dielmo and Ndiop and that observed during the Magal of Touba in groups of patients not all presenting respiratory symptoms [16,25,26], it is much lower than those observed in Dakar and other sites, with prevalences of up to 50% and 37.1% in children under the age of five (Supplementary Table S3) [14,15,27]. As previously reported in Senegal, adenovirus was also detected throughout the year, with varying amplitudes but no clear seasonality [27].

Influenza A (mostly H1N1) was the second most frequently detected virus (6.2%), while influenza B was detected in just one patient. Influenza A virus was significantly more frequent in patients with respiratory symptoms than those without (11.4% versus 2.9%). It was also more frequently detected in younger patients (under the age of ten). Overall, these prevalences are lower than those previously observed at other sites in Senegal (Supplementary Table S3) [14,15,24,25,26,28,29,30,31], with prevalence reaching 64.2% during an outbreak at the Touba Magal in September 2021 [32]. Vaccination against influenza is available, with the aim of reducing severe forms, complications, and the infectivity of vaccinated subjects compared to non-vaccinated subjects. The disease is characterized by outbreaks, usually seasonal, occurring worldwide every year, as well as occasional pandemics due to new subtypes [28]. This is why the different types of influenza (A or B) have different prevalences depending on the sites and years of the various studies. While data from the first epidemiological surveys of influenza in Senegal, carried out between 1996 and 2009 [33], showed that influenza activity always peaked during the rainy season (July–September), the most recent data [14,34], and our own, confirm that this is now less evidently the case in this subtropical area.

Although rhinovirus was the third most frequent virus, its overall prevalence (5%) was lower than those reported at other sites in Senegal, with prevalences in patients with influenza-like illness above 10%, most often between 20% and 30%, and occasionally up to 40% [13,14,15,24,25,26,29]. There was also no significant difference in prevalence between patients with fever and respiratory symptoms (6.5%) and those without (4.2%). Moreover, the prevalence was even lower than that (8.9%) recently observed in asymptomatic people in the village of Dielmo, located in the Fatick region of southern Senegal [25]. Outside of Senegal, several other studies have also reported the presence of rhinovirus in the respiratory secretions of between 12% and 22% of asymptomatic people [13]. Overall, rhinovirus was endemic throughout the year, with a peak showing greater amplitude during the rainy season, as previously reported [13].

The overall prevalence of RSV was 4%, and it was significantly more frequently observed in febrile patients with respiratory symptoms than in those without respiratory symptoms. No RSV was detected in asymptomatic people in Dielmo in the study by Diouf et al. [25], nor in that by Goumballa et al. in Touba [26]. Widely varying prevalences of RSV have been reported in patients in previous studies performed in Senegal depending on the sites, years, and age groups (Supplementary Table S3) [14,15,16,24,25,26,29,32]. The highest prevalences of approximately 16% to 20% were observed in young children in Dakar as well as in Keur Soce. Overall, the prevalence reported in our study appears to be lower than those reported previously. Measures taken in the wake of the COVID-19 pandemic may have reduced its spread. Further, as previously reported, the spread of RSV coincided with the rainy season [34].

Among the coronaviruses, SARS-CoV-2 was the most frequently detected with an overall prevalence of 4%. The highest peak observed was in January 2022, corresponding to the spread of the fourth wave of COVID-19 in Senegal [35]. Among the common coronaviruses, HCoV-HKU1 was the most frequent (1.8%), followed by HCoV-E229 (1.6%), HCoV-NL63 (1.2%), and HCoV-OC43 (0.6%). The overall prevalence of these four common coronaviruses (5.2%) is quite similar to those previously reported in Senegal, but the prevalence of each type is different according to the studies (Supplementary Table S3) [36,37]. No association with respiratory symptoms was observed with the different common coronaviruses. In the study by Diouf et al. in Dielmo, all samples positive for common coronaviruses were detected in asymptomatic people [25]. This was also the case in the Touba study by Goumballa et al. [38]. Our data on seasonality are consistent with those already observed in Senegal, with detection rates being highest from November to January for HCoV-OC43 and from September to January for HCoV-NL63, as well as sporadic circulation throughout the year for HCoV-229E. Data on HCoV-HKU1 are more scarce, and no clear seasonal pattern has yet been observed [36]. The overall prevalence of HPIV was 3.4%. Moreover, it was significantly higher in patients with respiratory symptoms (7.6%) than in those without (1%). No HPIV was detected in asymptomatic people in Dielmo in the study by Diouf et al. [25]. In our study, HPIV-1 was most frequently detected, followed by HPIV-3. HPIVs were exclusively detected in children under ten years old. However, in other studies in Senegal, HPIVs were also detected in older people, including people over the age of 50 [14]. Although the study by Dia et al. [14] reported that HPIV-1 and HPIV-3 were also the most frequent HPIVs, this was not the case in the study by Diouf et al. [25], where HPIV-2 and HPIV-3 were the most frequent, and HPIV-1 was not detected. Thus, the prevalence and type of HPIV depend on the study sites (Supplementary Table S3).

Among the viruses with an overall prevalence of less than 3%, metapneumovirus and bocavirus were mainly detected in children under the age of 11 and were both significantly correlated with the presence of respiratory symptoms. These values are within the range of those previously reported in Senegal [10,14,24,25,37,38]. While metapneumovirus shows seasonal variations, with higher circulation from late winter to early spring in temperate countries and during the rainy season in Senegal [39], data on the seasonality of bocaviruses are scarce. Bocaviruses circulate worldwide, all year round. However, a seasonal distribution has recently been reported in temperate countries, where infections are mainly detected in winter and spring [40,41]. In Senegal, a country with a mild subtropical climate, prevalence appears to be highest at the end of the dry season, when temperatures are lowest. Finally, to the best of our knowledge, this is the first time that parechovirus has been detected in nasopharyngeal samples in Senegal. It was exclusively observed in children under the age of six and with no difference regarding the presence or absence of respiratory symptoms, as observed for enterovirus.

The overall prevalence of bacteria in the Niakhar area was 64.8%, but they were significantly more frequently detected among patients with respiratory symptoms (80%) than those without (55.8%). *S. pneumoniae* was the predominant microorganism and was also significantly more frequent in patients with respiratory symptoms (51.9%) than in those without (28%). This prevalence is lower than that reported in Keur Soce (74%) in patients with symptoms suggestive of acute respiratory infection [24]. It is also much higher than that observed in an earlier study of children under the age of five with upper or lower airway infections in Dakar hospitals (17%), but the diagnostic strategies (culture versus qPCR) were not the same, making comparison even more difficult [15]. In their recent study in Dielmo, Diouf et al. also reported a prevalence of around 50% in symptomatic people, whereas the prevalence among asymptomatic people was 12.2% [25]. Although the incidence of invasive infections appears to have fallen with the introduction of pneumococcal vaccination [42], pneumococcus remains the main pathogen responsible for bacterial pneumonia [42]. However, according to the WHO (https://data.who.int/indicators/i/D45F91C, accessed on 3 June 2024), vaccination coverage with the third dose of pneumococcal conjugate vaccine in 1-year-old children in Senegal has improved by a total of 7%, from 81% in 2014 to 88% in 2022. The high prevalence of *S. pneumoniae* could also be linked to vaccine failure, with, among other causes, the circulation of serotypes that are not usually present. Indeed, the number of *S. pneumoniae* serotypes is high, and an emergence of non-vaccine serotypes has also been noted in several countries [43]. *H. influenzae* was also significantly more frequent in patients with respiratory symptoms (54.6%) than those without (24.5%). *S. pneumoniae* and *H. influenzae* were both present throughout the year, but with higher peaks during the dry season, while *C. propinquum* peaks were higher during the rainy season, even though it was also circulating all year round. The role of *C. propinquum* in respiratory infections is unclear and requires special attention. Indeed, very few cases of opportunistic respiratory infections have been reported with this bacterium [42].

Co-infections were significantly more frequent among patients with respiratory symptoms than those without (65.4% versus 32.9%). In addition to the consideration that primary infections by viral pathogens can predispose people to secondary bacterial infections, the different interactions between the resident microbiota and incoming pathogens, as well as interactions between viruses and respiratory bacteria, are thought to be at the root of the onset of respiratory symptoms [8,44,45,46,47,48]. However, we also noted that three patients with co-infections with five or even six pathogens did not present respiratory symptoms. This highlights one of the limitations of our study, as we have no information on patient follow-up, particularly regarding whether or not patients developed respiratory symptoms in the days following consultation. Furthermore, the presence of a virus and/or bacteria in a patient’s nasopharynx does not necessarily mean that these are the etiological agents of respiratory symptoms. It may also be the result of prolonged excretion of a pathogen that caused a previous infection, or of asymptomatic carriage.

As previously mentioned, multiplex PCR kits are costly, making large-scale use impractical in low- and middle-income countries. Moreover, the medico-economic impact of these kits on patient management is still being debated, with varying degrees of interest depending on age (newborns/children/adults), immune status (immunocompetent/immunocompromised), severity of symptoms, and the need to be hospitalized when possible, with accessibility and rapidity of results also being key factors [49,50,51]. The overall contributions expected from multiplex PCR tests beyond the cost of commercial kits are, in addition to the possibility of obtaining an etiological diagnosis, the reduction in unnecessary antibiotic prescriptions, the management of contagion, and sentinel surveillance of circulating respiratory pathogens. While the establishment of PCR laboratories in developing countries may seem difficult, it is nevertheless encouraged by the WHO (https://iris.who.int/bitstream/handle/10665/249549/9789290225317-Eng.pdf?sequence=5, accessed on 3 June 2024.).

Significant circulation of respiratory pathogens in febrile patients has been demonstrated in the Niakhar area in rural Senegal, including those preventable by vaccination such as *S. pneumoniae*. These epidemiological data complement those obtained in other areas of Senegal. They underline the importance not only of vaccination campaigns but also of their impact, raising the question of the serotypes of *S. pneumoniae* currently circulating in Senegal. In the future, although the medico-economic benefits of multiplex PCR are still debated, the availability of real-time etiological diagnostic tools with reduced costs should gain in importance in order to be able to adapt management as effectively as possible.

## Figures and Tables

**Table 1:** Prevalence of respiratory microorganisms from nasopharyngeal swabs in 500 febrile patients from the Niakhar area (Senegal) according to the presence or absence (w/o) of respiratory symptoms in 495 of them.

		495 Febrile Patients with Data on Respiratory Symptoms		
	500 Febrile Patients	185 with Respiratory Symptoms	310 w/o Respiratory Symptoms	
Microorganisms	Number of Positive Samples (Percentage)			*p*-Value
At least 1 microorganism	366 * (73.2%)	164 (89.0%)	197 (63.5%)	<0.001
At least one virus	193 * (38.6%)	105 (56.7%)	85 (27.4%)	<0.001
Adenovirus	59 (11.8%)	28 (15.3%)	31 (10.0%)	0.08
Influenza A	31 * (6.2%)	21 (11.4%)	9 (2.9%)	<0.001
Including A H1N1	20 * (4.0%)	16 (8.6%)	3 (1.0%)	<0.001
Influenza B	1 (0.2%)	1 (0.5%)	0	0.4
Influenza A and B	32 * (6.4%)	22 (11.9%)	9 (2.9%)	<0.001
Rhinovirus	25 (5.0%)	12 (6.5%)	13 (4.2%)	0.2
SARS-CoV-2	20 * (4.0%)	8 (4.3%)	10 (3.2%)	0.5
RSV	20 (4.0%)	12 (6.5%)	8 (2.6%)	0.03
Metapneumovirus	14 (2.8%)	10 (5.4%)	4 (1.3%)	0.007
HPIV-1	11 (2.2%)	9 (4.9%)	2 (0.6%)	0.003
HPIV-2	2 (0.4%)	1 (0.5%)	1 (0.3%)	1
HPIV-3	4 (0.8%)	4 (2.1%)	0	0.02
HPIV-4	0	0	0	NA
HPIV-1, -2, -3, and -4	17 (3.4%)	14 (7.6%)	3 (1.0%)	<0.001
Bocavirus	10 (2.0%)	7 (3.8%)	3 (1.0%)	0.04
HCoV-HKU1	9 (1.8%)	3 (1.6%)	6 (2.0%)	1
HCoV-E229	8 (1.6%)	2 (1.0%)	5 (1.6%)	1
HCoV-NL63	6 (1.2%)	3 (1.6%)	3 (1.0%)	0.7
HCoV-OC43	3 (0.6%)	0	3 (1.0%)	0.3
All common coronaviruses	26 * (5.2%)	8 (4.3%)	17 (5.5%)	0.6
Enterovirus	5 (1.0%)	3 (1.6%)	2 (0.6%)	0.4
Parechovirus	5 (1.0%)	3 (1.6%)	2 (0.6%)	0.4
At least one bacterium	324 * (64.8%)	148 (80.0%)	173 (55.8%)	<0.001
*S. pneumoniae*	184 * (36.8%)	96 (51.9%)	87 (28.0%)	<0.001
*H. influenzae*	179 * (35.8%)	101 (54.6%)	76 (24.5%)	<0.001
*C. propinquum*	130 * (26.0%)	49 (31.9%)	79 (25.4%)	0.8
*S. aureus*	15 (3.0%)	5 (2.7%)	10 (3.2%)	0.7
*S. pyogenes*	8 (1.6%)	3 (1.6%)	5 (1.6%)	1
*M. pneumoniae*	0	0	0	NA

* Data on respiratory symptoms are lacking for 5 patients. NA: Not applicable.

**Table 2:** Prevalence of respiratory microorganisms in 495 febrile patients from the Niakhar area (Senegal) according to age group and the presence or absence (w/o) of respiratory symptoms (RS).

	273 Patients from 1 mo to 5 y		87 Patients from 6 to 10 y		73 Patients from 11 to 20 y		62 Patients from ≥ 21 y	
	131 with RS	142 w/o RS	28 with RS	59 w/o RS	10 with RS	63 w/o RS	16 with RS	46 w/o RS
Microorganisms	Number of Positive (Percentage)		Number of Positive (Percentage)		Number of Positive (Percentage)		Number of Positive (Percentage)	
At least 1 microorganism	121 (92.4%)	102 (71.8%)	23 (82.1%)	41 (69.5%)	6 (60.0%)	32 (50.8%)	10 (62.5%)	26 (56.5%)
At least 1 virus	78 (59.5%)	45 (31.7%)	13 (46.4%)	17 (28.8%)	4 (40.0%)	13 (20.6%)	10 (62.5%)	10 (21.7%)
Adenovirus	24 (18.3%)	19 (13.3%)	1 (3.6%)	4 (14.8%)	2 (20.0%)	7 (11.1%)	1 (6.2%)	1 (2.2%)
Influenza A	16 (12.2%)	3 (2.1%)	5 (18%)	2 (3.4%)	0	3 (4.8%)	0	1 (2.2%)
Including A H1N1	11 (8.4%)	2 (1.4%)	5 (18.0%)	0	0	1 (1.6%)	0	0
Influenza B	0	0	0	0	0	0	1 (6.2%)	0
Influenzas A and B	16 (12.2%)	3 (2.1%)	5 (18.0%)	2 (3.4%)	0	3 (4.8%)	1 (6.2%)	1 (2.2%)
Rhinovirus	8 (6.1%)	9 (6.3%)	2 (7.1%)	2 (3.4%)	1 (10.0%)	1 (1.6%)	1 (6.2%)	1 (2.2%)
SARS-CoV-2	0	1 (0.7%)	5 (18.0%)	1 (1.7%)	2 (20.0%)	1 (1.6%)	3	5 (10.9%)
RSV	11 (8.4%)	5 (3.5%)	0	2 (3.4%)	0	0	1 (6.2%)	1 (2.2%)
Metapneumovirus	8 (6.1%)	2 (1.4%)	1 (3.6%)	1 (1.7%)	0	0	1 (6.2%)	1 (2.2%)
HPIV-1	8 (6.1%)	2 (1.4%)	1 (3.6%)	0	0	0	0	0
HPIV-2	0	1 (0.7%)	1 (3.6%)	0	0	0	0	0
HPIV-3	3 (2.3%)	0	1 (3.6%)	0	0	0	0	0
HPIV-4	0	0	0	0	0	0	0	0
HPIV-1, -2, -3, and -4	11 (8.4%)	3 (2.1%)	3 (10.8%)	0	0	0	0	0
Bocavirus	6 (4.6%)	2 (1.4%)	1 (3.6%)	0	0	0	0	1 (2.2%)
HCoV-HKU1	2 (1.5%)	4 (2.8%)	1 (3.6%)	2 (3.4%)	0	0	0	0
HCoV-E229	2 (1.5%)	2 (1.4%)	0	1 (1.7%)	0	0	0	2 (4.3%)
HCoV-NL63	1 (0.8%)	1 (0.7%)	1 (3.6%)	1 (1.7%)	0	0	1 (6.2%)	1 (2.2%)
HCoV-OC43	0	2 (1.4%)	0	1 (1.7%)	0	0	0	0
All common coronaviruses	5 (3.8%)	9 (6.3%)	2 (7.1%)	5 (8.5%)	0	0	1 (6.2%)	3 (6.5%)
Enterovirus	3 (2.3%)	2 (1.4%)	0	0	0	0	0	0
Parechovirus	3 (2.3%)	2 (1.4%)	0	0	0	0	0	0
At least one bacterium	113 (86.3%)	91 (64.1%)	20 (71.4%)	38 (64.4%)	6 (60.0%)	25 (39.7%)	9 (56.2%)	19 (41.3%)
*S. pneumoniae*	78 (59.5%)	45 (31.7%)	12 (42.8%)	21 (35.6%)	1 (10.0%)	11 (17.5%)	5 (31.2%)	10 (21.7%)
*H. influenzae*	80 (61.1%)	44 (31.0%)	13 (46.4%)	19 (32.2%)	1 (10.0%)	8 (12.7%)	7 (43.7%)	5 (10.9%)
*C. propinquum*	34 (26.0%)	37 (26.0%)	8 (28.6%)	18 (30.5%)	5 (50.0%)	13 (20.6%)	2 (12.5%)	11 (23.9%)
*S. aureus*	5 (3.8%)	4 (2.8%)	0	4 (14.8%)	0	1 (1.6%)	0	1 (2.2%)
*S. pyogenes*	1 (0.7%)	2 (1.4%)	1 (3.6%)	2 (3.4%)	1 (10.0%)	1 (1.6%)	0	0
*M. pneumoniae*	0	0	0	0	0	0	0	0

**Table 3:** Prevalence of co-infections in 185 febrile patients with respiratory symptoms and 310 without symptoms in the Niakhar area (Senegal).

495 Febrile Patients	185 Patients with Respiratory Symptoms	310 Patients without Respiratory Symptoms	
Co-Infections	Number of Co-Infections (Percentage)		*p*-Value
With 2 microorganisms	55 (29.7%)	56 (18.7%)	0.002
With 3 microorganisms	43 (23.2%)	33 (10.6%)	0.002
With 4 microorganisms	20 (10.8%)	10 (3.2%)	0.001
With 5 microorganisms	3 (1.6%)	2 (0.6%)	ns
With 6 microorganisms	0	1 (0.3%)	ns
Total	121 (65.4%)	102 (32.9%)	<0.001

ns = not significant.

## Data Availability

All data is available on reasonable request from the corresponding author.

## Supplementary Materials

The following supporting information can be downloaded at: https://www.mdpi.com/article/10.3390/pathogens13080655/s1, Table S1. PCR systems used in this study for real-time simplex PCR assays. Table S2. Prevalence and details of co-infections in 500 febrile patients from the Niakhar area (Senegal) and according to the presence or absence of respiratory symptoms in 495 of them. Table S3. Summary of the main previous studies on the prevalence of various respiratory micro-organisms in Senegal.
