# Supplementary material for: Prevalence of Respiratory Pathogens in Nasopharyngeal Swabs of Febrile Patients with or without Respiratory Symptoms in the Niakhar Area of Rural Senegal
Source: Pathogens. 2024 Aug 2;13(8):655. doi: 10.3390/pathogens13080655 (PMC11357141; doi:10.3390/pathogens13080655)
Supplement: Supplementary file 1 [file pathogens-13-00655-s001.zip › pathogens-2964740-supplementary/SupplementaryTable S1 2024_03_25.pdf]

Supplementary Table S1. PCR systems used in this study for real-time simplex PCR assays.

Micro-organisms	Targeted sequences	Names of primer and probe	Sequences	Amplicon size	References
Corynebacterium propinquum	DNA polymerase III subunit epsilon	Cprop_Dna3_MBF	TCACACTCACTGGCGAGTTC	116-bp	22
		Cprop_Dna3_MBR	GACTACCAGCACGGTGGTTT		
		Cprop_Dna3_MBP	6FAM-CCGGTTGTGGTCCGATATCGC		
Haemophilus influenzae	ompP1	Hinf_ompP1_F	CCTTACGTGCRGGTATKGCT	167-bp	17
		Hinf_ompP1_R	GTGAACTTTTTTGCCTTTTAAGTAAGC		
		Hinf_ompP1_P	6FAM-AGTGCTGCAATTCCAGATACCGATCGC		
Mycoplasma pneumoniae	P1	MPP1_F	CCGTTTTACTCGTGCCGCG	64-bp	18
		MPP1_R	GGGAGCGCTAACCCCCG		
		MPP1_P	6FAM-AGCGTGGTGTACTATGAGCAGTTGCAGC		
SARS-CoV-2	Small envelope protein-E-gene	E_Sarbeco_F1	ACAGGTACGTTAATAGTTAATAGCGT	113-bp	21
		E_Sarbeco_R2	ATATTGCAGCAGTACGCACACA		
		E_Sarbeco_P1	6FAM-ACACTAGCCATCCTTACTGCGCTTCG		
Staphylococcus aureus	*NucA*	Saur_NucA_F2	GTTGTGGATGGTGATACATTTATTGC	116-bp	20
		Saur_NucA_R2	CCAAATGGTTGTACAGGCGTATTC		
		Saur_NucA_P2	6FAM-AGGCTTATAGGGGTTGATACGCCAGAAACGG		
Streptococcus pneumoniae	*LytA*-CDC	lytA-CDC_F	ACGCAATCTAGCAGATGAAGCA	75-bp	19
		lytA-CDC_R	TCGTGCGTTTTAATTCCAGCT		
		lytA-CDC_P	6FAM-TGCCGAAAACGCTTGATACAGGGAG		
Streptococcus pyogenes	Hypothetical protein	Spyo_hypp_MBF	ACAGGAACTAATACTGATTGGAAAGG	125-bp	20
		Spyo_hypp_MBR	TGTAAAGTGAAAATAGCAGCTCTAGCA		
		Spyo_hypp_MBP	6FAM- AAATGTTGTGTTTTAGGCACTGGCGG		
