# Supplementary material for: Prevalence of Respiratory Pathogens in Nasopharyngeal Swabs of Febrile Patients with or without Respiratory Symptoms in the Niakhar Area of Rural Senegal
Source: Pathogens. 2024 Aug 2;13(8):655. doi: 10.3390/pathogens13080655 (PMC11357141; doi:10.3390/pathogens13080655)
Supplement: Supplementary file 1 [file pathogens-13-00655-s001.zip › pathogens-2964740-supplementary/SupplementaryTable S2 2024_03_25.pdf]

Supplementary Table S2. Prevalence and details of co-infections in 500 febrile patients from the Niakhar area (Senegal) and according to the presence or absence of respiratory symptoms in 495 of them.

		495 patients with data on RS	
	**500* patients**	**185* with RS**	**310* without RS**
**Micro-organisms**	**Number of positive (Percentage)**		
**Co-infections with 2 micro-organisms**	**113* (22.6%)**	**55 (29.7%)**	**56 (18.7%)**
S. pneumoniae - *H. influenzae*	39 (7.8%)	23 (12.4%)	16 (5.2%)
S. pneumoniae - *C. propinquum*	9* (1.8%)	2 (1.1%)	6 (1.9%)
H. influenzae - *C. propinquum*	9* (1.8%)	3 (1.6%)	5 (1.6%)
H. influenzae - Adenovirus	5 (1%)	4 (2.2%)	1 (0.3%)
C. propinquum - Adenovirus	5 (1%)	1 (0.5%)	4 (1.3%)
S. pneumoniae - Adenovirus	3 (0.6%)	1 (0.5%)	2 (0.6%)
S. pneumoniae - Influenza A	3 (0.6%)	3 (1.6%)	0
S. pneumoniae - SARS-CoV-2	3 (0.6%)	3 (1.6%)	0
S. pneumoniae - Human rhinovirus	2 (0.4%)	1 (0.5%)	1 (0.3%)
S. pneumoniae - RSV	2 (0.4%)	1 (0.5%)	1 (0.3%)
H. influenzae - HPIV-1	2 (0.4%)	2 (1.1%)	0
H. influenzae - Human rhinovirus	2 (0.4%)	1 (0.5%)	1 (0.3%)
C. propinquum - Influenza A	2 (0.4%)	2 (1.1%)	0
C. propinquum - Human rhinovirus	2 (0.4%)	0	2 (0.6%)
C. propinquum - SARS-CoV-2	2 (0.4%)	2 (1.1%)	0
S. pneumoniae - Metapneumovirus	1 (0.2%)	0	1 (0.3%)
S. pneumoniae - HPIV-1	1 (0.2%)	0	1 (0.3%)
S. pneumoniae - HCoV-NL63	1 (0.2%)	0	1 (0.3%)

S. pneumoniae - HCoV-229	1 (0.2%)	0	1 (0.3%)
S. pneumoniae - *S. aureus*	1 (0.2%)	0	1 (0.3%)
H. influenzae - RSV	1 (0.2%)	0	1 (0.3%)
H. influenzae - Metapneumovirus	1 (0.2%)	1 (0.5%)	0
H. influenzae - HPIV-3	1 (0.2%)	1 (0.5%)	0
H. influenzae - HCoV-NL63	1 (0.2%)	1 (0.5%)	0
H. influenzae - HCoV-229	1 (0.2%)	0	1 (0.3%)
H. influenzae - Influenza A	1 (0.2%)	0	1 (0.3%)
H. influenzae - SARS-CoV-2	1 (0.2%)	0	1 (0.3%)
C. propinquum - HCoV-229	1 (0.2%)	0	1 (0.3%)
C. propinquum - HCoV-HKU1	1 (0.2%)	0	1 (0.3%)
C. propinquum - HPIV-2	1 (0.2%)	0	1 (0.3%)
C. propinquum - RSV	1 (0.2%)	1 (0.5%)	0
C. propinquum - *S. pyogenes*	1 (0.2%)	0	1 (0.3%)
C. propinquum - *S. aureus*	1 (0.2%)	0	1 (0.3%)
SARS-CoV-2 - HCoV-NL63	1 (0.2%)	1 (0.5%)	0
HCoV-229E - Adenovirus	1 (0.2%)	1 (0.5%)	0
Adenovirus - *S. pyogenes*	1 (0.2%)	0	1 (0.3%)
Enterovirus - Metapneumovirus	1 (0.2%)	0	1 (0.3%)
RSV - Parechovirus	1 (0.2%)	0	1 (0.3%)
**Co-infections with 3 micro-organisms**	**77* (15.4%)**	**43 (23.2%)**	**33 (10.6%)**
S. pneumoniae - *H. influenzae* - Adenovirus	17 (3.4%)	9 (4.9%)	8 (2.6%)
S. pneumoniae - *H. influenzae* - *C. propinquum*	8 (1.6%)	5 (2.7%)	3 (0.1%)
S. pneumoniae - *H. influenzae* - Influenza A	6 (1.2%)	4 (2.2%)	2 (0.6%)

S. pneumoniae - *H. influenzae* - Human rhinovirus	5 (1%)	3 (1.6%)	2 (0.6%)
H. influenzae - *C. propinquum* - Influenza A	3 (0.6%)	1 (0.5%)	2 (0.6%)
S. pneumoniae - *H. influenzae* - HCoV-HKU1	2 (0.4%)	0	2 (0.6%)
S. pneumoniae - *C. propinquum* - RSV	2 (0.4%)	1 (0.5%)	1 (0.3%)
S. pneumoniae - *C. propinquum* - Human rhinovirus	2 (0.4%)	2	0
H. influenzae - *C. propinquum* - HPIV-3	2 (0.4%)	0	2 (0.6%)
S. pneumoniae - *H. influenzae* - RSV	2 (0.4%)	0	2 (0.6%)
S. pneumoniae - *H. influenzae* - Metapneumovirus	2 (0.4%)	0	2 (0.6%)
S. pneumoniae - *H. influenzae* - HPIV-1	1 (0.2%)	0	1 (0.3%)
S. pneumoniae - *H. influenzae* - HCoV-OC43	1 (0.2%)	0	1 (0.3%)
S. pneumoniae - *H. influenzae* - HCoV-NL63	1 (0.2%)	1 (0.5%)	0
S. pneumoniae - *H. influenzae* - Enterovirus	1 (0.2%)	1 (0.5%)	0
S. pneumoniae - *H. influenzae* - Bocavirus	1 (0.2%)	1 (0.5%)	0
S. pneumoniae - *S. aureus* - HCoV-HKU1	1 (0.2%)	0	1 (0.3%)
S. pneumoniae - *C. propinquum* - Adenovirus	1 (0.2%)	1 (0.5%)	0
S. pneumoniae - *C. propinquum* - HPIV-1	1 (0.2%)	1 (0.5%)	0
S. pneumoniae - Human rhinovirus - Enterovirus	1 (0.2%)	1 (0.5%)	0
S. pneumoniae - *C. propinquum* - Influenza A	1 (0.2%)	1 (0.5%)	0
S. pneumoniae - HCoV-HKU1 - HPIV-3	1 (0.2%)	1 (0.5%)	0
S. pneumoniae - *H. influenzae* - SARS-CoV-2	1 (0.2%)	1 (0.5%)	0
S. pneumoniae - *H. influenzae* - *S. aureus*	1 (0.2%)	1 (0.5%)	0
H. influenzae - Bocavirus - Adenovirus	1 (0.2%)	1 (0.5%)	0
H. influenzae - Metapneumovirus - Adenovirus	1 (0.2%)	1 (0.5%)	0
H. influenzae - RSV - Parechovirus	1 (0.2%)	1 (0.5%)	0

H. influenzae - HPIV-1 - Bocavirus	1 (0.2%)	1 (0.5%)	0
H. influenzae - *C. propinquum* - *S. aureus*	1 (0.2%)	0	1 (0.3%)
H. influenzae - *C. propinquum* - HPIV-2	1 (0.2%)	0	1 (0.3%)
H. influenzae - Influenza A - Influenza B	1 (0.2%)	1 (0.5%)	0
H. influenzae - SARS-CoV-2 - HCoV-229E	1*(0.2%)	*	*
C. propinquum - Bocavirus - Adenovirus	1 (0.2%)	1 (0.5%)	0
C. propinquum - Bocavirus - *S. aureus*	1 (0.2%)	0	1 (0.3%)
C. propinquum - Human rhinovirus - HCoV-NL63	1 (0.2%)	0	1 (0.3%)
C. propinquum - *S. aureus* - Influenza A	1 (0.2%)	1 (0.5%)	0
C. propinquum - RSV - Influenza A	1 (0.2%)	1 (0.5%)	0
**Co-infections with 4 micro-organisms**	**30 (6%)**	**20 (10.8%)**	**10 (3.2%)**
S. pneumoniae - *H. influenzae* - *C. propinquum* - Metapneumovirus	3 (0.6%)	3	0
S. pneumoniae - *H. influenzae* - *C. propinquum* - Adenovirus	2 (0.4%)	2 (1.1%)	0
S. pneumoniae - *H. influenzae* - *C. propinquum* - Influenza A	2 (0.4%)	1 (0.5%)	1 (0.3%)
S. pneumoniae - *H. influenzae* - Adenovirus - Bocavirus	2 (0.4%)	1 (0.5%)	1 (0.3%)
S. pneumoniae - *H. influenzae* - Adenovirus - Human rhinovirus	2 (0.4%)	1 (0.5%)	1 (0.3%)
S. pneumoniae - *H. influenzae* - Bocavirus - Metapneumovirus	2 (0.4%)	1 (0.5%)	1 (0.3%)
S. pneumoniae - *H. influenzae* - *C. propinquum* - SARS-CoV-2	1 (0.2%)	0	1 (0.3%)
S. pneumoniae - *H. influenzae* - *C. propinquum* - Human rhinovirus	1 (0.2%)	1 (0.5%)	0
S. pneumoniae - *H. influenzae* - *C. propinquum* - HCoV-HKU1	1 (0.2%)	1 (0.5%)	0
S. pneumoniae - *H. influenzae* - *C. propinquum* - *S. pyogenes*	1 (0.2%)	0	1 (0.3%)
S. pneumoniae - *H. influenzae* - *C. propinquum* - Enterovirus	1 (0.2%)	1 (0.5%)	0
S. pneumoniae - *H. influenzae* - Adenovirus - Influenza A	1 (0.2%)	0	1 (0.3%)
S. pneumoniae - *H. influenzae* - RSV - Influenza A	1 (0.2%)	1 (0.5%)	0

S. pneumoniae - *H. influenzae* - RSV - Human rhinovirus	1 (0.2%)	1 (0.5%)	0
S. pneumoniae - *H. influenzae* - HPIV-1 - Influenza A	1 (0.2%)	1 (0.5%)	0
S. pneumoniae - *H. influenzae* - *S. pyogenes* - HCoV-HKU1	1 (0.2%)	0	1 (0.3%)
S. pneumoniae - *H. influenzae* - *S. aureus* - Adenovirus	1 (0.2%)	0	1 (0.3%)
S. pneumoniae - *H. influenzae* - RSV - SARS-COV-2	1 (0.2%)	0	1 (0.3%)
S. pneumoniae - *H. influenzae* - Influenza A - SARS-CoV-2	1 (0.2%)	1 (0.5%)	0
S. pneumoniae - *C. propinquum* - HCoV-NL63 - SARS-CoV-2	1 (0.2%)	1 (0.5%)	0
S. pneumoniae - *S. pyogenes* - HCoV-HKU1 - SARS-CoV-2	1 (0.2%)	1 (0.5%)	0
H. influenzae - *C. propinquum* - RSV - Metapneumovirus	1 (0.2%)	1 (0.5%)	0
H. influenzae - Parechovirus - RSV - Metapneumovirus	1 (0.2%)	1 (0.5%)	0
**Co-infections with 5 micro-organisms**	**5 (1%)**	**3 (1.6%)**	**2 (0.6%)**
S. pneumoniae - *H. influenzae* - *C. propinquum* - SARS-CoV-2 - RSV	1 (0.2%)	0	1 (0.3%)
S. pneumoniae - *H. influenzae* - *C. propinquum* - Bocavirus - HPIV-1	1 (0.2%)	1 (0.5%)	0
S. pneumoniae - *H. influenzae* - *C. propinquum* - Human rhinovirus - Enterovirus	1 (0.2%)	0	1 (0.3%)
S. pneumoniae - *H. influenzae* - Parechovirus - RSV - HPIV-1	1 (0.2%)	1 (0.5%)	0
S. pneumoniae - *H. influenzae* - *S. aureus* - *S. pyogenes* - RSV	1 (0.2%)	1 (0.5%)	0
**Co-infections with 6 micro-organisms**	**1 (0.2%)**	**0**	**1 (0.3%)**
S. pneumoniae - *H. influenzae* - Parechovirus - RSV - HCoV-OC43 - Human rhinovirus	1 (0.2%)	0	1 (0.3%)
**Total**	**226* (45.2%)**	**121 (65.4%)**	**102 (32.9%)**

RS= respiratory symptoms. * Data on respiratory symptoms are lacking for 5 patients.
